# Peers as OSCE assessors for junior medical students – a review of routine use: a mixed methods study

**DOI:** 10.1186/s12909-019-1898-y

**Published:** 2020-01-16

**Authors:** Simon Schwill, Johanna Fahrbach-Veeser, Andreas Moeltner, Christiane Eicher, Sonia Kurczyk, David Pfisterer, Joachim Szecsenyi, Svetla Loukanova

**Affiliations:** 1 Department of General Practice and Health Services Research, University Hospital Heidelberg, Heidelberg, Germany; 2 Competence Center Assessment in Medical Education, University of Heidelberg, Heidelberg, Germany

**Keywords:** Testing/assessment, Peer assessment, General practice, Primary care, Education, Communication skills, Problem-based learning, OSCE (objective structured clinical examination)

## Abstract

**Background:**

Peer-assisted learning is well established in medical education; however, peer tutors rarely act as assessors for the OSCE. In the compulsory, near-peer teaching programme covering basic medical skills at the University of Heidelberg, peer tutors serve as assessors on a formative OSCE. This study aimed to investigate the feasibility and acceptance of peer assessors and to survey the perceived advantages and disadvantages of their use.

**Methods:**

In 2016 and 2017 all OSCE peer assessors (third to sixth-year medical students) and all of the peer-assessed students in 2017 (second-year-medical students) were invited to participate in a survey. Both groups were asked to complete a tablet-based questionnaire immediately after the OSCE. Peer assessors were asked to rate eight statements and the peer-assessed students to rate seven statements on a five-point Likert scale. Both were asked to comment on the advantages and disadvantages of peer-assessors.

**Results:**

Overall, 74 of 76 peer assessors and 307 of 308 peer-assessed students participated in the study. 94% (67/74) of peer assessors and 90% (276/307) of the peer-assessed group thought that it is important to have peer tutors as assessors. Of the peer assessors, 92% (68/74) felt confident in giving structured feedback during the OSCE and 66% (49/74) felt they had improved their teaching skills. Of the peer-assessed students, 99% (306/307) were satisfied with their peers as OSCE assessors and 96% (292/307) considered the peer feedback during the OSCE as helpful. The participants mentioned structural benefits, such as lower costs, and suggested the quality of the OSCE was higher due to the use of peer assessors. The use of peer assessors was found to be beneficial for the learners in the form of high-quality feedback and an overall reduction in stress. Furthermore, the use of peer assessors was found to be beneficial for the peer assessors (improved teaching and clinical skills).

**Conclusion:**

From a learner’s perspective, the use of peer assessors for a formative OSCE that is part of a near-peer teaching program aimed at junior medical students is favourable for all. A broad implementation of peer assessment in the formative OSCE should be encouraged to investigate effects on quality and stress-reduction.

## Background

The Objective Structured Clinical Examination (OSCE) aims to examine the medical student’s clinical competence [1]. In general, an OSCE is comprised of several rotations (=stations), at which students demonstrate their practical skills, for instance in examination techniques and anamnesis (history-taking) [2–4]. The reliability and educational value of an OSCE is high [5], but the organisational burden and costs of administering one are high due to the need for human resources and the time involved [6]. An OSCE can be performed in formative or graded version [2, 7, 8]. Sometimes feedback is also given during the OSCE [9].

Peer-assisted learning, also known as (near-)peer tutoring or (near-)peer teaching, represents an educational approach in which more experienced students teach younger and less experienced ones [10, 11]. The use of peer teachers is beneficial not only for the students who acquire teaching skills and improve their organisational competencies, but also for the universities since it enables teaching in a small-group setting at a manageable cost [10, 12]. In general, peer teaching is already established in the health professions, especially in nursing education, with good results [13, 14]. However, the literature lacks reports of the formal integration of near-peer teaching programmes into medical curricula. In a scoping review by Khan et al., peer assessors seem to benefit from improvement in feedback and teaching skills [9]. There are only few reported studies available on peer tutors who serve as assessors in a formative OSCE [2, 4, 9, 15–19].

At the University of Heidelberg in Germany, there is a continuous, near-peer teaching programme during the first two pre-clinical years of medical school. This compulsory programme (AaL^*plus*^) imparts basic medical skills in history-taking, physical examination and practical skills to all medical students (approximately 320 per year) and culminates in a formative OSCE at the end of the second year. As of 2013, peer tutors in AaL^*plus*^ have been deployed as assessors for the OSCE [20].

Little is known about the peer tutors as assessors in the formative OSCE from the perspective of either the peer assessors or the peer-assessed students.

The aim of this study was to investigate the feasibility and acceptance of using peer assessors for the formative OSCE in the conventional medical curriculum and to survey student perspectives on peer assessors in the OSCE, including the advantages and disadvantages.

## Methods

### Study design

We conducted a cross-sectional survey of medical students in the role of peer assessor or peer-assessed.

### Setting and participants

This study was conducted at the Medical Faculty of Heidelberg University, Germany. The university’s medical curriculum, HeiCuMed (*Heidelberger Curriculum Medicinale*), is 6 years in length and divided into two pre-clinical and four clinical years and is offered to about 320 students per year. During the first two pre-clinical years, the continuous, near-peer teaching programme, AaL^*plus*^, teaches basic medical skills in history-taking, physical examination and practical skills such as venepuncture. Embedded within the longitudinal general practice curriculum, AaL^*plus*^ is organised by the Department of General Practice and Health Services Research [20]. Participation in AaL^*plus*^ is mandatory for all medical students.

The main goal of AaL^*plus*^ is to ensure basic medical skills in history-taking and clinical examination with practical training by the end of the second year. To accomplish this, there is a continuous core curriculum during the first 2 years of medical school (Table 1).

**Table 1:** The AaLplus curriculum in the first and second years of medical study

Basis skills				
Semester	History taking	Physical examination	Practical skills	Problem-oriented learning
1	Introduction to physician/patient communication	Introduction to physical examination, Examination of the musculoskeletal system	Hand disinfection, Venepuncture	1: History taking
2	Introduction to Anamnesis	Examination of the thorax and abdomen	Voluntary training in venepuncture	
3	Seven dimensions of a symptom	Neurological examination	-	2: Literature searches, 3/4: situations in general practice
4	History taking	Examination of the thyroid, pulse and lymphatic system, Refresher: physical examination from head to toe	Formative OSCE	

The history-taking tutorial is taught using “standardised patients” (trained amateur actors), who are regularly supervised to ensure an authentic performance and a high quality of feedback. Students learn clinical examination by performing examinations on each other using standardised checklists, available as the “Heidelberg Standards of Examination” (*Heidelberger Standarduntersuchungen*) [21]. Practical skills such as venepuncture are taught using mannequins and with each other on a voluntary basis after obtaining the necessary consents. Problem-oriented learning takes place in small groups.

To ensure the high quality of AaL^*plus*^, students provide evaluations and feedback at the end of every year. Peer tutors collect additional responses. Peer tutors, themselves, also have the option to evaluate and discuss problems. The AaL^*plus*^ programme was successfully implemented in 2011 and has been constantly updated since 2014, as shown in Table 1.

One distinctive feature of AaL^*plus*^ is that the tutorials and the OSCE at the end of the second year are held solely by peer tutors. Peer tutors have to pass three basic tutorials in which they are taught general teaching and moderation skills, team leadership and conversational techniques by educational professionals, general practitioners and psychologists. Moreover, they must successfully complete specific training in problem-oriented learning and other subject-specific training, such as clinical examination of the heart, lungs and abdomen every year. In addition, experienced peer tutors referred to as “trainers” offer refresher seminars at the beginning of each year to the other tutors. In general, all peer tutors are supported and supervised by general practitioners or psychologists during their first teaching session, and as needed or requested.

New peer tutors are selected on the basis of grades, previous clinical experience and motivation. Experienced peer tutors participate in the selection and training of new peer tutors. Becoming an AaL^*plus*^ peer tutor is highly competitive, with five times as many applicants than positions each year.

### OSCE

At the end of the fourth pre-clinical semester, a formative (mandatory but ungraded) OSCE is taken, based on feedback. The OSCE is organised in four stations (history-taking, two stations for clinical examination, and venepuncture) with a rotation time of 5 min for each station, 3 min. for peer feedback, and one additional minute to switch to the next station. During the OSCE, peer tutors serve as peer assessors and are supervised by medical staff. The peer assessors are medical students at a more advanced clinical semester who have completed training in teaching, as well as clinical techniques, and have successfully served as peer tutors within the AaL^*plus*^ programme. Peer assessors rate the peer-assessed students using tablet-based checklists (tOSCE Programme [22, 23], on which they fill in scores during the observation. The scoring helps to provide feedback to the peer-assessed group, but is not used for grading. Later, the peer-assessed students may check their scores via an online platform.

### Data collection

At the end of the 2016 and 2017 academic years, all peer tutors serving as OSCE assessors were invited to participate in an evaluation. In 2017 all second-year medical students were also invited to participate in the evaluation. The peer assessor and the peer assessed evaluations were both tablet-based and obtained directly after the last OSCE session. In 2016, the OSCEs were administered on June 14 and 18, and in 2017 on May 19, 20 and 26.

### Outcome measures

The peer assessors were asked to complete an evaluation composed of an eight-item questionnaire (using a five-point Likert scale: I agree completely, I agree, neutral, I disagree, I disagree completely). The questionnaire was embedded into the general course evaluation conducted by the medical school. It was developed by a team of experienced researchers (SS, CE, SK, JS, SL) and on the basis of a comprehensive literature analysis, which showed several priority areas of interest, such as feedback in the formative OSCE and improvement of teaching skills [12, 24, 25]. The questionnaire was previously tested using a think-aloud technique on former AaL^*plus*^ peer tutors (Additional files 1 and 2). The evaluation underwent constant revision over the first 2 years of the programme. The items in the quantitative component focused on self-preparation for the OSCE, the role of the tutors as assessors, and on improvements in their feedback skills. The questions asked of the peer-assessed students focused on the feedback, acceptance of the near-peer tutors as assessors in general, the students’ tutorship in AaL^*plus*^ and the knowledge gained throughout the process. The main component in order to address the beneficial effects from the learners point of the were the open-text-sections: In the qualitative component of the questionnaire comprised of open-text-sections, both peer assessors and the peer-assessed group were asked: “What are the advantages of using peer tutors as OSCE assessors?” and ““What are the disadvantages of using peer tutors as OSCE assessors?”

### Data analysis

Sociodemographic datasets were analysed descriptively using percentages, median rate and range. Quantitative data were analysed using an exploratory approach and descriptive statistics using SPSS. Qualitative content analysis by Mayring was used to analyse the open-text sections [26, 27]. Main categories were pre-assigned dividing advantages and disadvantages into peer-assessed benefits, peer-assessor benefits, and general benefits. Original data in German were analysed independently by two researchers experienced in qualitative research (SS, JFV). In a second step, codes and subcategories, as well as the English translations, were discussed with a third researcher (SL) until consensus was reached.

## Results

### Socio-demographic data

Overall, *n* = 74 peer assessors (2016 and 2017) and *n* = 308 peer-assessed students (2017) participated in the study.

Table 2 shows the socio-demographic data of 38 peer assessors in 2017. A total of 59% of the peer assessors were male and 41% were female. The mean age of a peer assessor was 23 years, with a range between 20 and 32 years. With 42%, most of the peer assessors were in their fourth year of medical school, 26% were in their third year and 18% were in their final year of medical school. One peer tutor was a second-year student, reflecting his exceptionality as a registered nurse. Normally, the status of peer tutor might be granted in the third year of medical school after successful performance on the First State Medical Examination. The mean years of experience as a peer tutor were 2 years, with a range of 1–5 years. At the end of the academic year, 37% of the peer tutors were novices with 1 year of experience as a peer tutor. The number of peer students serving as OSCE assessors for the first time was 53%. Only one peer tutor had 5 years of experience as an OSCE assessor. The overlap of peer assessors in 2016 and 2017 was *n* = 15.

**Table 2:** Sociodemographic data of peer-assessors (senior medical students 3rd-6th year)

	n		*n*	%	Median [range^a^]
Gender	(*n* = 34)	female	14	41%	
		male	20	59%	
Mean age	(*n* = 38)				23 yrs. [20-32]
Rotation year	(*n* = 38)				4th yr. [2-6]
		2nd	1	3%	
		3rd	10	26%	
		4th	16	42%	
		5th	4	11%	
		6th = final	7	18%	
Experience as peer-tutor	(*n* = 35)				2 yrs. [1-5]
		Beginner (1st yr.)	13	37%	
		Advanced (2+)	22	63%	
Experience as OSCE-assessor	(*n* = 36)				1st yr. [1-5]
		Beginner (1st yr.)	19	53%	
		Advanced (2+)	17	47%	

*yr.* year, *yrs.* years

^a^full range

The socio-demographic data of 307 peer-assessed students were obtained at the end of the 2017 academic year. A total of 51% were female and 49% were male. The mean age of the peer-assessed students was 21 years and the range was 18 to 34 years. Of this group, 30% already possessed advanced qualification in a health profession (e.g. dentist, paramedic, physiotherapist, registered nurse, technician) or another field before starting medical school.

### Quantitative data analysis

**Fig. 1:**
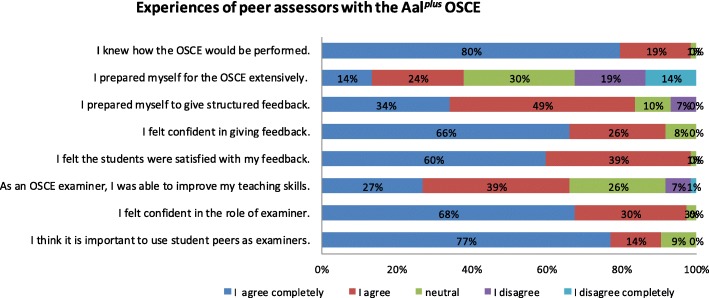
Experiences of peer assessors in the OSCE of the AaL^plus^ programme in 2016 and 2017 (*n* = 74)

Nearly all peer assessors (99%) agreed or completely agreed that they understood how the OSCE would be performed. Twenty-eight of 74 of peer assessors (38%) agreed or completely agreed that they had previously prepared themselves for the OSCE (Fig. 1). Twenty-five peer assessors (33%) did not prepare themselves. Sixty-eight of 74 peer tutors (92%) felt confident in giving a structured feedback to the peer-assessed students and 83% had prepared themselves to give structured feedback prior to the OSCE. A total of 99% felt that the students were satisfied with the feedback given; 66% (49/74) agreed or completely agreed that acting as an OSCE assessor had improved their teaching skills. A total of 68% (50/74) felt comfortable with being an assessor, and 91% (67/74) agreed or completely agreed that it is important to use peers as assessors.

**Fig. 2:**
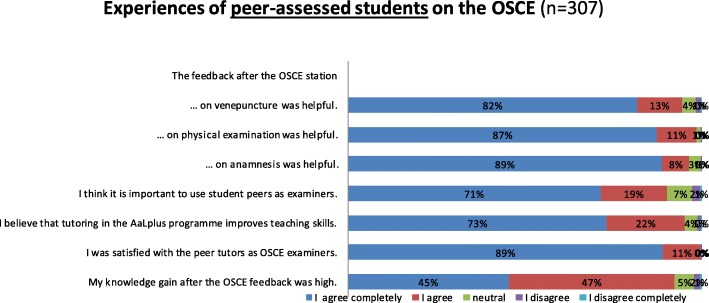
Peer-assessed students’ evaluation after the OSCE of the AaL^plus^ programme in 2017 (*n* = 307)

Three hundred seven questionnaires were obtained from the peer-assessed group and analysed (response rate 99.7%). A total of 92% of the peer-assessed students agreed or completely agreed that they gained substantial knowledge from taking the OSCE (Fig. 2). 306 of 307 peer-assessed students (99%) were satisfied with the peer tutors as assessors in the OSCE. 95% agreed or completely agreed that tutorship in AaL^*plus*^ programme improves teaching skills. 276 of 306 peer-assessed students (90%) found it important to have near-peer tutors as assessors. Over 95% of the peer-assessed group agreed or completely agreed that the feedback after the OSCE stations on history-taking, physical examination and venepuncture was helpful.

In comparison, 95% of the peer-assessed students agreed or completely agreed that tutorship in the AaL^*plus*^ programme improves teaching skills, whereas 66% of the peer assessors agreed or completely agreed that they could improve their teaching skills as assessors. 90% of the peer-assessed group found it important to have peer tutors as assessors versus 91% of the peer assessors who agreed or completely agreed that having peers as assessors is important. A total of 99% of the peer assessors had the impression that the peer-assessed students were satisfied with the feedback they had given and, altogether, over 95% of the peer-assessed students agreed or completely agreed that the feedback they received was helpful.

### Qualitative data analysis

We obtained qualitative data from 74 peer assessors and 307 peer-assessed students. The advantages of using peer tutors as assessors in the OSCE are summarised in Table 3. The disadvantages of using peer tutors as assessors in the OSCE are presented in Table 4. The responses to the question, “What are the advantages of using peer tutors as OSCE assessors?” were divided into three categories: (1) benefits for the AaL^*plus*^ programme, (2) benefits for the peer assessors, and (3) benefits for the peer-assessed students. The answers to the question, “What are the disadvantages of using peer tutors as OSCE assessors?” were categorised according to the same categories.

**Table 3:** Advantages of peer-tutors as assessors in the OSCE: Categories used to code the content of qualitative data (peer-assessors *n* = 74, peer-assesses *n* = 307)

	Peer assessors		Peer-assessed	
Category	Subcategory	Code /description	Subcategory	Code /description
For AAL^*plu*^ (in general)	Reduction of costs	- Peer assessors are cheaper than trained doctors/professionals.	Reduction of costs	- Peer assessors are cheaper than trained doctors/professionals. - Peer assessors are available in high numbers. - Peer assessors support doctors/professionals (economy of time) - Relaxed atmosphere increases the efficiency of the OSCE.
	Quality control	- Peer assessors as OSCE assessors learn which teaching and learning material can be adjusted because they have previously taught the students.	Quality control	–
	Quality improvement	- Peer assessors receive more training in teaching and giving feedback than the average academic lecturer. - Peer assessors know exactly what was taught in the programme because they teach it. As a result, they are much more attuned to student expectations (e.g. OSCE checklists). - Peer assessors have better knowledge of the curriculum and content of AaL^*plus*^. - Peer assessors can more easily estimate a student’s level of knowledge. - Peer assessors do know how to answer student’s questions better.	Quality improvement	- Peer assessors receive more training in *the perfect physical examination* than the average academic lecturer. - Peer assessors know what has been previously taught in the programme. - Peer assessors may be more interested in keeping the OSCE and the programme up to date. - Learning is more important than the examination. Peer assessors are up-to-date and refer to current standards.
For peer assessors:	Benefits for own studies	- By assuming an assessor’s point of view, peer assessors prepare and train for their own future OSCEs/examinations. - Peer assessors receive a sense of transparency regarding their own OSCEs.	Benefits for own studies	–
	Improvement of feedback skills	- Peer assessors gain experience in giving structured feedback. - Peer assessors improve their skills in giving feedback. - Peer assessors learn how to structure and give feedback properly.	Improvement of feedback skills	–
	Personal benefits	- Peer assessors are enabled to take responsibility.	Personal benefits	- Peer assessors improve their social skills.
	Improvement of clinical skills	- While testing the student, peer assessors reinforce their own skills (e.g. physical examination of the heart and lungs).	Improvement of clinical skills	- While testing the student, peer assessors reinforce their own skills (e.g. physical examination of the heart and lungs).
	Improvement of teaching skills	- Peer assessors receive feedback themselves about their general performance as a teacher during the previous 2 years.	Improvement of teaching skills	- Peer assessors improve their competencies as an assessor.
For peer-assessed:	High-quality feedback	- Students accept peer assessors’ feedback more easily since they are at eye level. - Students receive feedback from the peer assessors who have previously trained them. - Students receive better feedback from peer assessors because they are more mindful of the students’ situations and perspectives. - Peer assessors take more time while giving feedback. - Peer assessors are more open to student questions. - Peer assessors can give better recommendations.	High-quality feedback	- Students accept peer assessors’ feedback more easily since they are at eye level. - Students take peer assessors’ feedback more seriously. - Students receive a better feedback from peer assessors because they are more mindful of the students’ situations and perspectives. - Peer assessors are more open to student questions. - Peer assessors can give better recommendations because they better understand why a student is making mistakes. - Peer assessors focus more on the student while giving feedback (*student-focused feedback*). - Students find it easier to ask questions about the feedback. - Peer assessors have better knowledge of students’ perspectives and feelings. - Peer assessors know the pitfalls regarding the learning content.
For peer-assessed:	Additional valuable information	- Peer assessors provide valuable information on further OSCEs. - Peer assessors inform about the typical OSCE pitfalls from a student perspective.	Additional valuable information	- Peer assessors provide more valuable and more helpful recommendations. - Peer assessors can remove fears of failure, both on the OSCE and in medical studies. Peer assessors give feedback for future OSCEs and for medical studies.
	Stress reduction	- Students show lower levels of stress when tested by peer assessors in general. - Peer assessors are more trustworthy. - Students have a reduced inhibition threshold to share information. - Peer assessors can remove apprehensions	Stress reduction	- Students show lower levels of stress when tested by peer assessors in general. - Students have a reduced inhibition threshold to share information. - Students can focus on the exam more easily with less distraction by lecturers.
	Comfortable atmosphere	There is a flatter hierarchy. - Students feel more comfortable if tested by peers. - Students accept peer assessors’ feedback more easily in a comfortable atmosphere.	Comfortable atmosphere	- Peer assessors establish a comfortable atmosphere. - Comfortable because of same eye level - Comfortable atmosphere results in fun while learning. - Peer assessors behave more cooperatively. - Peer assessors have better access to students. - Personal relationships result in fun while learning.
	Appreciation	–	Appreciation	- Peer assessors are more focused and are not bored during the examination.
	Knowledge gain during the OSCE	- Peer assessors create a learning atmosphere in the OSCE. - Students feel free to ask assessors for adjustments and, as a result, better understand their personal performance	Knowledge gain during the OSCE	–

**Table 4:** Disadvantages of peer-tutors as assessors in the OSCE: Categories used to code the content of qualitative data (peer assessors *n* = 74, peer-assesses *n* = 307)

	Peer assessors		Peer-assessed	
Category	Subcategory	Code /description	Subcategory	Code /description
For AAL^*plus*^ (in general)	Reduced obligation	- Peer assessed students might not take the OSCE seriously enough. - Peer assessors have a lack of authority compared to lecturers and might not be accepted. - Hard to maintain conditions of a compulsory examination.	Reduced obligation	- Peer-assessed students might not take the OSCE seriously enough. - Peer assessors have a lack of authority compared to lecturers and might not be accepted.
	Reduced professionalism	- Peer assessors might not be as objective as doctors/professionals. - Relaxed atmosphere misleads peer-assessed/assessor relationship.	Reduced professionalism	- Peer-assessed students are more likely to know peer assessors from another or a negative situation. - Peer assessors are less professional. -Lack of objectivity
	Benignity	- Peer assessors might be more benign as they often know the peer-assessed students personally.	Benignity	- Peer assessors might be more benign as they often know the peer-assessed students personally.
	- Strictness	–	Strictness	- Peer assessors might be stricter.
For peer assessors:	*not mentioned*		*not mentioned*	
For peer-assessed:	Little experience as assessor	- Peer assessors might have less experience in lecturing. - Peer assessors are less self-confident.	Little experience as assessor	- Peer assessors might have less experience in lecturing. - Peer assessors do not have a doctor’s/professional’s speciality.
	Reduced medical skills	- Peer assessors have little clinical experience. - Peer assessors have less clinical skill than lecturers and cannot give such good advice.	Reduced medical skills	- Peer assessors have little clinical experience. - Peer assessors do not know physical examination in *reality.* - Peer assessors may be less experienced than peer-assessed students (e.g. who have previous paramedic training) - Peer assessors cannot estimate clinical relevance.
	Reduced value of feedback	- Peer assessors’ feedback might be less relevant technically. - Lecturers give feedback of higher quality.	Reduced value of feedback	- Doctors/professionals have better knowledge of what is important in the future.
	Less clinical/medical knowledge	–	Less clinical/medical knowledge	-Peer assessors have a lack of medical knowledge. - Peer assessors spread ignorance.
	Personal relationship	–	Personal relationship	- Peer-assessed students may disgrace themselves because they know the peer assessor. - Setting might be awkward.

Both the peer-assessed students and their peer assessors identified structural benefits for the AaL^*plus*^ programme, specifically the reduction of costs. The peer assessors suggested that the use of peer assessors is a quality control within AaL^*plus*^ [“*Because they have previously taught the students, peer tutors as OSCE assessors learn which teaching and learning materials can be adjusted.*”]. The peer assessors and peer-assessed students agreed that deploying peer tutors as OSCE assessors improves quality because peer assessors undergo more in-depth training than the professional staff and that because they have served as peer tutors within the 2 years prior to the OSCE, peer assessors have better knowledge of the course content. Furthermore, peer assessors claimed that they can easily judge a student’s level of knowledge and that they understand how to answer students’ questions [“*We are more familiar with the curricular material and can often better understand the problems students are having.*”]. Peer-assessed students mentioned that peer assessors are often more aware of and refer to current standards.

The benefits for peer assessors include the improvement of teaching, feedback and clinical skills. They also gain personal benefits such as the improvement of social skills and the opportunity to assume responsibility. Finally, the experience of serving as an assessor provides peer assessors with useful knowledge regarding their own upcoming examinations and, especially, OSCEs [“*sense of transparency about own OSCEs*”].

In regard to the benefits for peer-assessed students, they along with peer assessors often mentioned the provision of high-quality feedback, the creation of a pleasant atmosphere and an overall reduction in stress. First, both groups suggest that peer-assessed students accept feedback from peer assessors more easily because they are on the same level academically and they have established a personal relationship. Second, it was reported that the peer assessors’ feedback is of a higher quality because peer assessors are mindful of the students’ personal situations and take more time to provide feedback. Their recommendations are more useful because they personally understand what students are going through [“*student-focused feedback*”]. Third, “*peer feedback is better because students find it easier to ask follow-up questions about the feedback and therefore get clearer takeaways.*”

Another main advantage mentioned by nearly all in the peer-assessed group was the overall reduction in stress; nearly every peer-assessed student reported that peer assessors create a comfortable atmosphere. This was due to the presence of the peer assessors and the more minimal difference in academic and social hierarchy. Some peer-assesses even considered the pleasant atmosphere as a fun learning environment [“*To the students the OSCE is an unusual and new way of examination. Therefore, I like the idea to avoid pressure at the first time. I think, it eases the situation and increases the benefit of learning*”].

In addition, peer assessors and peer-assessed students often highlighted valuable information regarding future OSCEs and the knowledge gained during the OSCE [“*It is easier to focus on the OSCE when you are not irritated by a professional assessor. Additionally, peer assessors in their fourth or fifth year of study better understand why you perform badly and can give better advice.*”]. One student wrote, “*Peer assessors can remove the fear of failure, for both the OSCE and medical study.*” Finally, the close social and academic standing between peer-assessed students and peer assessors resulted in behaviour that improves the overall atmosphere of the OSCE and enabled high levels of knowledge transfer *[“Peer assessors are more focused (than professional staff) and are not bored during the examination*”].

The main disadvantages reported by both groups were categorised as: (1) structural disadvantages and (2) disadvantages for the peer-assessed students. Neither peer-assessed students, nor the peer assessors mentioned any disadvantages for the peer assessors.

The structural disadvantages reported by the two groups included the limited professional competencies of peer assessors and a possible lack of objectivity on the part of peer assessors. In general, both groups identified less obligation and limited professionalism on the part of peer assessors. Less experience as assessors, a lower level of medical skill and limited clinical knowledge were all identified as disadvantages to using peer tutors as assessors. Furthermore, peer assessors may not be as objective as professional staff due to the relaxed atmosphere and personal relationship. Furthermore, personal relationships between peer assessed students and peer assessors can lead to awkward situations [“*Students may disgrace themselves because they know the peer assessors*”]. For peer assessors this could be caused by a difficulty maintaining an atmosphere appropriate to a compulsory examination, as was mentioned by one peer assessor.

The disadvantage for the peer-assessed students is that they might not take the OSCE seriously enough. Finally, 65 peer-assessed students (22%) and 15 peer assessors (20%) responded that there are no disadvantages to having peer tutors act as assessors for the OSCE.

## Discussion

In this study we found an overwhelming approval of using peers as OSCE assessors by both the learners and the peer assessors. We found that the implementation of peer assessment in the compulsory medical curriculum is feasible. From the learner’s perspective, peer assessed students benefit greatly from the use of peer assessors because the feedback is detailed and precise, making it more helpful than feedback from professional staff. Peer assessors benefit personally from improved clinical, social and teaching skills. Finally, from learner’s point of view peer assessors contribute to a profound reduction in stress within the formative OSCE, which is felt to be pivotal for individual success in learning.

### Medical students favour peer-examiners

To the best of our knowledge this is the first study to report on broad implementation of a formative OSCE with peer-assessors in routine. We have more than 5 years of experience in the mandatory curriculum and more than 1500 medical students have passed the formative peer-led OSCE within the last 5 years. Our results indicate that medical students generally accepted peers as examiners in a formative OSCE. First published in 2010, a mock OSCE was found to be beneficial for student nurses [28]. In 2014, Young et al. published an extracurricular educational intervention in which fourth-year medical students prepared a mock OSCE for their near-peers [29] raising questions of objectivity [30]. In 2017, medical students described an approach of a peer-led mock OSCE they had planned and administered [31]. Updated in 2018, Lee et al. published their experiences with a mock OSCE in a trend article [32]. Our data shows that from a learner’s perspective peer assessors are beneficial.

### Peer assessors can improve their clinical, social and teaching skills

The benefits for peer assessors, including improvement in feedback and teaching skills and the consolidation of knowledge through teaching and administering examinations, has also been summarised in a scoping review by Khan et al. [9]. Those authors concluded that participating in OSCEs promote learning for both peer assessors and peer-assessed students. Burgess et al. [24] investigated the role of final-year medical students as OSCE assessors for second- year medical students. The peer assessors reported the review of clinical skills and knowledge as “a way to assess, review and develop their own knowledge and clinical skills” [24]. Providing feedback to peers is seen as an effective learning experience for students [33]. In regard to the CanMEDS competencies, peer tutoring enhances the role of the scholar [34, 35]. Interestingly, the concepts of peer teaching and peer assessing have not yet been shown to be effective or increase teaching skills. A randomised trial with physiotherapy students showed dissatisfaction with near-pear teaching [36]. In summary, the beneficial effects on teaching skills are only seen in qualitative studies, indicating a great need for studies proving the beneficial effects of peer assessment.

### Peer assessment: improvement of quality?

Interestingly, both peers assessed students and peer assessors claimed that there is an improvement in quality connected to the use of peer assessors compared to professional staff. Surprisingly, the few studies on peer tutors as OSCE assessors found only little difference between peer tutors as OSCE assessors compared to professional staff as OSCE assessors [2, 17, 24, 36–38]. The inter-rater reliability of students as assessors was shown to be good in one study from 2007 with moderate to high agreement with teaching staff [17]. Another study did not find significant differences between dental student assessors and professional assessors [36]. However, in our experience, inter-rater reliability in the OSCE remains challenging. Therefore, further studies and more comparisons between experienced peer assessor ratings and professional examiner ratings are needed to explore relative inter-reliabilities in the OSCE. Additionally, quality of the OSCE is not only about inter-rater reliability. The suggested differences in the quality of feedback between peer assessors and professional staff needs to be investigated and either proven or refuted whereas other factors of structural, process and outcome quality should be considered. Notably, a recent study could not prove students, who has participated in a formative OSCE perform better in subsequent OSCEs, although previous studies described self-reported benefits from participation in student-led MOSCEs [39].

### .Peer assessment could be implemented in routine curriculum

Finally we have shown that peer assessors in a formative OSCE at the conclusion of a mandatory near-peer teaching programme imparting basic medical skills could be successfully implemented in the curriculum of a large medical school with 320 students annually. The hidden curriculum of the formative OSCE at the end of the second year is to familiarise students with the OSCE’s format and decrease negative expectations or even fears of the OSCE in subsequent years of medical school. Our study indicates that we have fulfilled this goal. We have learned that if students have been previously trained as peer tutors, they are able to perform successfully as OSCE assessors. Other medical schools should be encouraged to implement peer assessments in near-peer teaching programs for junior medical students in routine.

### Limitations

In this explorative study we showed that deploying peers as assessors remains a successful practice. One limitation of this study is that the close relationship between the peer assessors and the peer-assessed students may have resulted in less critical responses from peer-assessed students relating to the performance of peer tutors as OSCE assessors (effect of benignity). Second, this study was implemented as part of the routine evaluation. An extended study design, such as interviewing key actors, might have supplied further information. Finally, many participants are convinced that utilising peers as assessors reduces the level of stress for peer-assessed students, whereas the formative nature of the OSCE itself might also reduce stress levels. Therefore, the belief in the beneficial effect of stress reduction caused by the use of peer assessors may be a biased point of view and the amount of stress-reduction by peers as assessors could be overemphasised. Future studies and broad use of peers as assessors in qualifying OSCE are necessary to improve our understanding on the effect of stress-reduction.

## Conclusion

The use of peer assessors in the OSCE in a near-peer teaching programme for junior medical students is feasible and could be successfully implemented into medical curricula. Furthermore, over 90% of the peer assessors and peer-assessed students believe it is important to have peers as assessors. From an organisational point of view, deploying peer assessors enables the implementation of compulsory formative OSCE training for a high volume of medical students while reducing the impact on faculty resources. For peer assessors, the experience of acting as OSCE assessors provides valuable knowledge and improves physician competencies (role of professional scholar). Peer-assessed students respect peer assessors and appreciate the high quality of feedback they provide and the relaxed atmosphere during their first and formative OSCE. In contrast to this clear and self-confident point of view of the learner’s, it remains to us to prove or refute the equivalence or even improvement of quality as well as the effect of stress-reduction by student led peer assessment in the (mock) OSCE .

## Supplementary information

**Additional file 1.** Questionnaire for peer assessors.

**Additional file 2.** Questionnaire for peer-assessed-students.

## Data Availability

The datasets used and/or analysed during the current study are available from the corresponding author upon reasonable request.

## Abbreviations

AaL: *Anamtomie am Lebenden*
OSCE: Objective Structured Clinical Examination

## References

[1] Wass V, Van der Vleuten C, Shatzer J, Jones R (2001). Assessment of clinical competence. Lancet..

[2] Moineau G, Power B, Pion A-MJ, Wood TJ, Humphrey-Murto S (2011). Comparison of student examiner to faculty examiner scoring and feedback in an OSCE. Med Educ.

[3] Harden RM, Gleeson FA (1979). Assessment of clinical competence using an objective structured clinical examination (OSCE). Med Educ.

[4] Iblher P, Zupanic M, Karsten J, Brauer K (2015). May student examiners be reasonable substitute examiners for faculty in an undergraduate OSCE on medical emergencies?. Medical Teacher.

[5] Sloan David A., Donnelly Michael B., Schwartz Richard W., Felts Janet L., Blue Amy V., Strodel William E. (1996). The Use of the Objective Structured Clinical Examination (OSCE) for Evaluation and Instruction in Graduate Medical Education. Journal of Surgical Research.

[6] Kelly M, Murphy A (2004). An evaluation of the cost of designing, delivering and assessing an undergraduate communication skills module. Medical Teacher.

[7] Rolfe I, McPherson J (1995). Formative assessment: how am I doing?. Lancet.

[8] Hill DA, Guinea AI, McCarthy WH (1994). Formative assessment: a student perspective. Med Educ.

[9] Khan R, Payne MWC, Chahine S (2017). Peer assessment in the objective structured clinical examination: A scoping review. Med Teacher.

[10] Topping KJ (1996). The effectiveness of peer tutoring in further and higher education: a typology and review of the literature. High Educ.

[11] Whitman NA, Fife JD. Peer Teaching: To Teach Is To Learn Twice. ASHE-ERIC Higher Education Report No. 4, 1988: ERIC; 1988.

[12] Burgess A, McGregor D, Mellis C (2014). Medical students as peer tutors: a systematic review. BMC Med Educ.

[13] Irvine S, Williams B, McKenna L (2018). Near-peer teaching in undergraduate nurse education: an integrative review. Nurse Educ Today.

[14] Irvine S, Williams B, McKenna L (2017). How are we assessing near-peer teaching in undergraduate health professional education? A systematic review. Nurse Educ Today.

[15] Hall S, Harrison CH, Stephens J, Andrade MG, Seaby EG, Parton W (2018). The benefits of being a near-peer teacher. Clin Teach.

[16] Cushing AM, Westwood OMR (2010). Using feedback in a formative objective structured clinical examination. Med Educ.

[17] Chenot J-F, Simmenroth-Nayda A, Koch A, Fischer T, Scherer M, Emmert B (2007). Can student tutors act as examiners in an objective structured clinical examination?. Med Educ.

[18] Melcher P, Zajonz D, Roth A, Heyde C-E, Ghanem M. Peer-assisted teaching student tutors as examiners in an orthopedic surgery OSCE station – pros and cons. GMS Interdisciplinary Plastic and Reconstructive Surgery DGPW. 2016 07/14;5:Doc17. PubMed PMID: PMC4950802. 10.3205/iprs000096 PMC4950802 27500078

[19] Siddiqui S, Siddiqui S, Mustafa Q, Rizvi AF, Hossain IT. The benefits of a peer-assisted mock PACES. The Clinical Teacher.n/a-n/a. 10.1111/tct.12658 28612515

[20] Ledig T, Eicher C, Szecsenyi J, Engeser P. AaLplus – history taking and physical examination a course for preclinical medical students. ZFA. 2014;90.

[21] Nikendei C, Ganschow P, Groener JB, Huwendiek S, Köchel A, Köhl-Hackert N, et al. “Heidelberg standard examination” and “Heidelberg standard procedures” – Development of faculty-wide standards for physical examination techniques and clinical procedures in undergraduate medical education. GMS J Med Educ 2016 08/1533(4):Doc54. PubMed PMID: PMC5003136. 10.3205/zma001053 PMC5003136 27579354

[22] Thamburaj AJ, Brass K, Herrmann M, Junger J. 8th meeting of the medical assessment consortium UCAN: "Collaborative Perspectives for Competency-based and Quality-assured Medical Assessment". GMS Zeitschrift fur medizinische Ausbildung. 2015;32(4):Doc37. PubMed PMID: 26483850. Pubmed Central PMCID: 4606488. 10.3205/zma000979 PMC4606488 26483850

[23] UCAN. Available from: https://www.ucan-assess.org/tosce/?lang=en. Accessed 14 Dec 2019.

[24] Burgess A, Clark T, Chapman R, Mellis C (2013). Senior medical students as peer examiners in an OSCE. Med Teacher.

[25] Burgess Annette, Black Kirsten, Chapman Renata, Clark Tyler, Roberts Chris, Mellis Craig (2012). Teaching skills for students: our future educators. The Clinical Teacher.

[26] Krippendorff K. Content analysis. An introduction to its methodology. Beverly 26 Hills: Sage; 1980.

[27] Mayring P (2007). Qualitative Inhaltsanalyse [content analysis].

[28] Paul F (2010). An exploration of student nurses' thoughts and experiences of using a video-recording to assess their performance of cardiopulmonary resuscitation (CPR) during a mock objective structured clinical examination (OSCE). Nurse Educ Pract.

[29] Young I, Montgomery K, Kearns P, Hayward S, Mellanby E (2014). The benefits of a peer-assisted mock OSCE. Clin Teach.

[30] Fletcher A, Day R (2015). A peer-led mock OSCE improves subsequent performance: what about objectivity?. Med Teach..

[31] Emery AW, Rose-Innes E (2018). Benefits of a peer-led mock-OSCE. Med Teach.

[32] Lee CB, Madrazo L, Khan U, Thangarasa T, McConnell M, Khamisa K (2018). A student-initiated objective structured clinical examination as a sustainable cost-effective learning experience. Med Educ Online.

[33] Kernan WN, Quagliarello V, Green ML (2005). Student faculty rounds: a peer-mediated learning activity for internal medicine clerkships. Medical Teacher.

[34] Canada. RCoPaSo. The CanMEDS 2015 Phyisician Competency Framework. http://www.royalcollege.ca/rcsite/canmeds-e 2005 [cited 2018 28.02.2018].

[35] Homberg A, Hundertmark J, Krause J, Brunnée M, Neumann B, Loukanova S. Promoting medical competencies through a didactic tutor qualification 730 programme – a qualitative study based on the CanMEDS Physician 731 Competency Framework. BMC Med Educ 2019 19,187. 10.1186/s12909-019-1636-5 PMC6549272 31164127

[36] Sevenhuysen Samantha, Skinner Elizabeth H, Farlie Melanie K, Raitman Lyn, Nickson Wendy, Keating Jennifer L, Maloney Stephen, Molloy Elizabeth, Haines Terry P (2014). Educators and students prefer traditional clinical education to a peer-assisted learning model, despite similar student performance outcomes: a randomised trial. Journal of Physiotherapy.

[37] Ogden G., Green M., Ker J. (2000). The use of interprofessional peer examiners in an objective structured clinical examination: Can dental students act as examiners?. British Dental Journal.

[38] Reiter Harold I., Rosenfeld Jack, Nandagopal Kiruthiga, Eva Kevin W. (2004). Do Clinical Clerks Provide Candidates with Adequate Formative Assessment during Objective Structured Clinical Examinations?. Advances in Health Sciences Education.

[39] Madrazo Lorenzo, Lee Claire Bo, McConnell Meghan, Khamisa Karima, Pugh Debra (2019). No observed effect of a student-led mock objective structured clinical examination on subsequent performance scores in medical students in Canada. Journal of Educational Evaluation for Health Professions.

