# Supplementary material for: Peers as OSCE assessors for junior medical students – a review of routine use: a mixed methods study
Source: BMC Med Educ. 2020 Jan 16;20:17. doi: 10.1186/s12909-019-1898-y (PMC6966898; doi:10.1186/s12909-019-1898-y)
Supplement: Supplementary file 1 — Additional file 1. Questionnaire for peer assessors. [file 12909_2019_1898_MOESM1_ESM.docx]

### Supplemental Data – Questionnaire for peer assessors

### Schwill et al: Peers assessors for junior medical students – a review of routine use: A mixed methods study

| **Peer examiner** | |
| --- | --- |
|  | I agree completely  I agree  neutral  I disagree  I disagree completely |
| I knew how the OSCE would be performed. |  |
| I prepared myself for the OSCE extensively. |  |
| I prepared myself to give structured feedback. |  |
| I felt confident in giving feedback. |  |
| I felt the students were satisfied with my feedback. |  |
| As an OSCE examiner, I was able to improve my teaching skills. |  |
| I felt confident in the role of examiner. |  |
| I think it is important to use student peers as examiners. |  |

| What are the advantages of using peer tutors as OSCE examiners? | *In the form of comments, please:* |
| --- | --- |

| What are the disadvantages of using peer tutors as OSCE examiners? | *In the form of comments, please:* |
| --- | --- |
