# Supplementary material for: Peers as OSCE assessors for junior medical students – a review of routine use: a mixed methods study
Source: BMC Med Educ. 2020 Jan 16;20:17. doi: 10.1186/s12909-019-1898-y (PMC6966898; doi:10.1186/s12909-019-1898-y)
Supplement: Supplementary file 2 — Additional file 2. Questionnaire for peer-assessed-students. [file 12909_2019_1898_MOESM2_ESM.docx]

### Supplemental Data – Questionnaire for students

### Schwill et al: Peers assessors for junior medical students – a review of routine use: A mixed methods study

| **Student** | |
| --- | --- |
|  | I agree completely  I agree  neutral  I disagree  I disagree completely |
| The feedback after the OSCE station on venepuncture was helpful. |  |
| The feedback after the OSCE station on physical examination was helpful. |  |
| The feedback after the OSCE station on anamnesis was helpful. |  |
| I think it is important to use student peers as examiners. |  |
| I believe that tutoring in the AaL*^plus^* programme improves teaching skills. |  |
| I was satisfied with the peer tutors as OSCE examiners. |  |
| My knowledge gain after the OSCE feedback was high. |  |

| What are the advantages of using peer tutors as OSCE examiners? | *In the form of comments, please:* |
| --- | --- |

| What are the disadvantages of using peer tutors as OSCE examiners? | *In the form of comments, please:* |
| --- | --- |
